# The British Hospitals Association: Fourth Annual Conference at Oxford

**Published:** 1913-07-05

**Authors:** 


### THE BRITISH HOSPITALS ASSOCIATION.

### FOURTH ANNUAL CONFERENCE AT OXFORD.

### Full Report of the Discussion.

The annual Conference of this Association at the famous University City of Oxford, under the Presidency of Sir William Osler, Bart., the Regius Professor of Medicine, was from every standpoint a distinct success, and the weather, fortunately, was propitious. By the kind permission of the Vice-Chancellor of the University the meetings were held in the spacious Examination Schools, and a visit of inspection was paid to the Radcliffe Infirmary and its Pathological Department, now nearly finished.

The Mayor of Oxford (Councillor S. Hutchins) extended to the Conference, on behalf of the citizens, a hearty and cordial welcome to the city, and expressed his satisfaction that the meetings would be held in such a spacious building, which had been so kindly placed at the disposal of the Association for the purpose. He knew but little of medicine, he said, but so long as Sir William Osler was in the chair and pioneering the ship of affairs medical in Oxford, he knew that everything possible would be done to mitigate suffering and suppress the ravages of the diseases which were so prevalent among communities. He trusted that the visit would not only prove instructive to members, but that much would be found to entertain, so that a pleasurable memory would go with them when the Conference was over.

The Vice-Chancellor of the University said he wished to echo, on behalf of the University, what the Lord Mayor had said for the citizens. In this matter, as in so many others, the City and the University were in hearty co-operation. Everyone must recognise not only the importance of hospital and all medical work, but that its importance was increasing, among other reasons because of the Insurance Act, which the Conference was to discuss at its first sitting. It was a source of satisfaction to him that the delegates were to visit the Radcliffe Infirmary. The debates which were to ensue would no doubt be profitable, and he hoped it would also be found that Oxford was a pleasant place in which to spend spare time.

Sir William Osler, on behalf of the Association, expressed thanks for the kind words of welcome by the Mayor and the Vice-Chancellor, and then proceeded to address the meeting. His address appears on page 411.

### DISCUSSION ON THE NATIONAL HEALTH INSURANCE ACT.

Dr. D. J. Mackintosh, M.V.O., Glasgow, thanked Sir William Osler for his most instructive address, though some of the points in it he did not understand. Sir William said that the sooner the voluntary principle was done away with in the hospitals the better. That was probably correct to a point, but it had yet to be shown that the voluntary principle had been a failure. If it had been shown to have failed he would say: Do not nibble at it, but make it a State matter right away. Only two clauses in the Act dealt with hospitals. Clause 12 said that, by making an arrangement, hospitals might be paid some of the money which went to the dependants of the insured. But an arrangement must be made. If the hospitals were merely to get the sum left over in regard to insured persons who had no dependants it certainly would not enable the hospital doors to be kept open. The country must either go to the State for support or the voluntary system must receive more enthusiasm than in the past. In Glasgow the workmen contributed to three hospitals £20,000, and inquiry showed that the introduction and working of the Insurance Act during six months had not made more than £80 difference, so that the artisan was not materially reducing his support. Working men asked, when the Insurance Act came into force, what part of the man's medical benefit would be taken by the hospital. The principle he had gone on—and he thought it was the practice in all the infirmaries in Glasgow—was that they took none of the sickness benefit; that went to the sick man, and the hospital had not any bargain, or come to any agreement with the Government or Local Government Board in the meantime. As the Insurance Act was about to be amended, he deprecated coming to any decision that day which could go out as the view of the Association until the matter had received the careful consideration of the Council. So long as the industrial classes continued their support of hospitals why should those institutions expect part of the sickness benefit? As the Act was framed it was intended that that should go to the sick man and his dependants. The question was a large one and seriously affected medical education; for if Britain had not well-equipped general hospitals, laboratories, and every modern convenience, she could not compete with other countries or do the best for the patients. In Germany patients were taken in at fees ranging from 12s. to 2s. 6d. a day, which they or their societies paid. There was sure to be some malingering, and where could it be so well detected as at a hospital? It was not likely to be "spotted" by the panel doctor going his rounds. Even if new buildings should be erected they would require to be maintained, and he urged that no agreement should be made with any governing body or with any approved society to take any sum in the meantime until it was seen what the cost of upkeep would be. There should be another six months' trial of the Act. Clause 12, providing that approved societies should be allowed to make a weekly payment practically for work done, was as near to State control as one could conceive. To take in patients at so much per week and give them a receipt was simply handing over the hospitals to the State right away. He would be glad to know that this discussion would be limited to the way in which during the last six months the Insurance Act had affected the hospitals, and what were the prospects for the future.

The President said the Germans paid a considerable sum to the hospitals.

Sir Henry Burdett:—

I think Dr. Mackintosh has largely misapprehended the first point made by Professor Osler, which was that the country and county hospitals should give up relying upon the provision of voluntary help which had proved inadequate to supply the needs of all classes of the community. With regard to the question of paying patients in hospitals, no one knew better than the President how well that system could be made to work efficiently and at a profit when carried on throughout on business principles. In this country, though reputed to be slow to move, people were alert to test new proposals. In 1877 the London press was full of the question of the admission of pay-patients and adequate provision for the "poor-rich" who wanted surgical and medical care. Yet this requirement at that date, when surgery was in its infancy, was as nothing compared to its claim to-day. Money was provided by the public at that date—1877—for a pay-hospital, and it had had a successful existence ever since, so that it had been rebuilt, and was still going on well. It admitted none but pay-patients, charging them as little as possible, but sufficient to keep it a sound business concern. He supported Sir William Osler's plea for pay-wards in the voluntary hospitals for all classes, and hoped one result of this Conference would be that throughout the country at the hospitals the question would be seriously discussed, and that there would soon be paying pavilions graded to meet the requirements of the various members of the community.

With regard to National Insurance, that had come to stay, and he formed that conclusion from a study of the German system, and despite political utterances. Its unpopularity was due to the fact that the wisest and most knowledgeable people had not been consulted, and the most experienced administrators' advice had not been taken. But at that Conference the speakers were not political; they were hospital people concerned with the treatment and care of the sick, who had to conduct their business under considerable difficulties. They were tired of the absence of statesmanship in high places; and were of opinion that such complicated measures should not be submitted and passed through the Legislature until they had been thoroughly thought out and made practicable. Hasty legislation caused a great deal of avoidable suffering and disappointment. He agreed with Dr. Mackintosh that they must wait until the Act had come fully into action, and see how the system worked, while continuing to accept patients under the old conditions. Patients were still being received, because the hospital authorities recognised that their first duty was towards the sick, and, whatever changes might be made by the Legislature, the first thing they must do was to see that the hospital provision should be adequate to the demands upon it. Hospital managers and officials must recognise what the Act was going to do for all the people, and therefore they must not only wait, but also prepare. In London alone it was estimated that there would ultimately be 2½ million insured persons, and at present probably from half a million to three-quarters of a million had not yet been brought under the Insurance Act. So that the pressure on hospital beds now was nothing like what it possibly would be, because the people now hanging back would from their needy circumstances prove to be those who would most need hospital care. So in London alone 100,000 sick people would require medical attendance every week. Actuarial calculation showed that, on the lowest computation, 10,000 beds would require to be constantly occupied by insured persons on this basis. Yet only 10,000 beds were available in the voluntary hospitals in London. More hospital beds would therefore be required, but the problem was as to where they were to come from. He believed an additional 10,000 beds would ultimately be needed, and 5,000 of those within the next two years. It must be remembered that the panel doctors were really recruiting agents, bringing under hospital care many severe cases who had not hitherto been shepherded in voluntary hospitals. If the Insurance Act fulfilled its mission, its great ultimate result should be to add to the maximum earning power of the working classes, by seeking out and securing medical treatment for those who needed it, and so to relieve and restore them as quickly as possible. The longer the needed treatment was delayed the greater would be the cost of making the patient well. The Act made no provision to pay for the cost of treatment of any patient in a hospital; that was the extraordinary omission from the measure which had astonished the world. He had been asked, when abroad, whether this "nation of shopkeepers" had lost its business sense. He was recently in a London hospital catering for 400,000 inhabitants, in which there were under 150 beds, whereas 500 beds were needed, and the site permitted of a building large enough to contain them. Liverpool had set a very good example, for Liverpool had lately taken power under a recent Act to make grants to the Liverpool Royal Infirmary, to St. Paul's Ophthalmic Hospital, and to another institution to enable them to erect new wards and provide additional buildings. He thought the demand for increased accommodation for insured persons in hospitals as it was required ought to be met by the Government agreeing to lend the money to defray the cost of new buildings wherever the need arose, the loan being at 3 per cent., with a sinking fund of 1 per cent. This interest, etc., for all such loans should be a first charge upon the *pro rata* payments for the maintenance of the patients, both insured patients and pay-patients, contained in the new pavilions which had been so erected. When the German system of insurance first came into force there were in that country practically few hospitals, in the British sense, at all; there were military hospitals, some clinical hospitals, and a few charitable institutions. National insurance in Germany had brought a new hospital system to that country. That system was municipal, and some of these new hospitals were more complete and more up to date than any he had seen elsewhere in the world. They received from the insured person a payment on the lowest scale of from 2s. 6d. to 3s. a day, and this was paid by the approved societies, not by the Government. The patients in these German hospitals were graded, and the payments ranged from 3 to 12 marks, or more, accordingly. But one of the latest German contributions on the subject advocated a reversion to the English or voluntary system, so that by means of a benevolent fund the new charges for hospitals on the rates could be lightened. That showed the need for educating the public, after the managers of the voluntary hospitals had agreed as to whether they would introduce the pay system; and, secondly, the necessity of setting their house in order and bringing pressure to bear on the Government as required to provide money on the Liverpool principle for the provision and maintenance of buildings. Several members of the Cabinet were in favour of the plan which he had suggested. It merely meant pledging the national credit to enable the hospitals throughout the country to maintain the voluntary principle and extend the area of their present activities. There was one bogey at the back of this proposed system—namely, that, if there were some such system of loans and repayments, there must necessarily be State control. That he totally denied, and the Government would have no claim to such control, for they would only be providing the capital sum necessary for the hospitals to be maintained and extended to the necessary extent to enable them to cater for insured persons and paying patients. Without some such businesslike co-operation National Insurance, as an actuarial undertaking, could not continue to exist.

Mr. Lupton, Leeds General Hospital:—

I think that all who are present will agree it would have been much better if the Insurance Act had been framed after consultation with those who had the management of the country's hospitals. What had happened was exactly what had been forecasted. Its effect had been to place hospital managers in a position of great difficulty, and it would be profitable to consider to-day how those difficulties could be overcome. While there was a diminution since the passing of the Act in the number of out-patients at hospitals, and to some extent also in the number of casualty cases, the number of insured persons had increased. He did not agree with Sir Henry Burdett that the best way was to get large buildings erected; the great problem was as to the maintenance of the necessary buildings. Either it could be required that all insured persons coming to the hospital should be paid for by the State or by the municipality, and if a common demand were made for that, it would be granted, because it was impossible to allow people requiring this treatment to go on without it. He felt sure that as time went on the voluntary contributions to hospitals would diminish. It would be a good plan to take the patient in and treat him, and at the end of the time send in the bill for maintenance to the authorities at 27s. 6d. per week, or some such sum. If that were done, and the voluntary contributions could be kept up by the rest of the people, there might be a chance of making both ends meet. The system mentioned by Sir William Osler of having both paying patients and voluntary patients commended itself to him; yet he felt the difficulty which had been pressed upon him by his own medical men as to the fate of the younger medical men, when perhaps not more than two guineas per week was charged, which really was only the maintenance charge, and nothing remained for the doctor. He believed the solution would be by having both pay and voluntary patients.

Mr. W. G. Carnt, Superintendent, Royal Infirmary, Manchester:—

I do not think that at present the hospitals are likely to get a *pro rata* payment from the Government for insured persons. But if an effort were made to get certain clauses in the Act amended, he believed the hospitals would benefit considerably; they would at any rate recover from the State the equivalent of what they would lose in annual subscriptions in consequence of the introduction of the Insurance Act. Though some hospitals had not found much reduction in contributions since the passing of the Act, yet there had been an absence of that increase which otherwise would probably have occurred. Sections 12 and 21 were the only clauses in the Insurance Act dealing with hospitals. At the Manchester Royal Infirmary it had been the custom for many years to interview every patient admitted, or, if he was too ill, to question the friends as to the circumstances of the family and their ability to contribute a small sum towards maintenance. This had been so successful that in the year before the Act was introduced these payments totalled £4,000. Since the Act came into force that sum had increased, because patients with dependants were now better off than formerly. When a patient was found to have no dependants, his society and his membership number were ascertained, and he was asked to sign a printed form saying he wished his sickness benefit to be paid to the hospital, because there was a clause which said the patient should be consulted if possible. In many cases the society replied that they would pay, and when the patient was discharged an account was sent, and the money was received. £102 had been received from that source. But some societies would not agree to this; the money went into the funds of the society, and did not benefit the patient. That was a matter which Parliament might quite fairly be asked to alter; the "may" should be converted into "shall." Clause 21 of the Act said that approved societies might give hospitals a donation or subscription. Was it the experience of any of those present that they had done so? It was not his own experience. He would like to see it enacted that the societies should be made to enter into an agreement with the hospitals if the hospitals desired it.

Dr. Mackintosh desired to point out that Circular A.S. 94, dated June 1913, said that the society must, where practicable, first ascertain the member's wishes in the matter; and must either pay the whole sum direct to the dependants, or, if he thought fit, to maintenance, in whole or in part, payment of rent, and providing with necessaries. Then, if there were no dependants, if the member were an inmate of any of the other institutions mentioned in the second paragraph of the circular, the society might apply the sum in providing the member with surgical appliances where necessary, or might otherwise expend it for his benefit. In many cases, the circular proceeded, the society might think it desirable to use the money to help the member over the difficulties incidental to his illness, such as by paying his rent or providing him with necessaries.

Sir Henry Burdett said he had gone into the question of the refusal of the societies to fall in with the suggestion of paying the sick benefit of persons without dependants to the hospitals, and it was a matter of business. The friendly societies and the insurance societies were in competition, and they declined to give this money to the hospitals because their competitors kept the money back, and the man was told it would be used for his benefit after he came out of hospital. Therefore, in actual practice this clause must be largely a dead letter.

Mr. J. H. Cooke, Albert Infirmary, Winsford, expressed his gratitude for Sir William Osler's able suggestions, but it must be remembered that this country differed a good deal from Canada and the United States, and we were very largely free from the corruption existing in the United States and the Colonies.

The President said he knew there was no corruption in America in connection with the large general hospitals.

Mr. J. H. Cooke:—

I certainly hold the view that there is no need to trouble about State and municipal intervention in connection with our hospitals: the people were already taxed to the uttermost, and no Government would face putting on an additional burden. A point which had not been mentioned was that under the Public Health Act every local authority had power to contribute towards the maintenance of the sick in the district. In his district there were only 11,000 inhabitants, yet they had contributed out of the rates to the Royal Eye Hospital at Manchester. It was surcharged by the district auditor, but it went before the law officers of the Crown, who decided that the town had the right to make the contribution. Though the amount was small, the procedure established a very important principle. If members went about the matter in the proper way, he did not think they would lose much under the Insurance Act. If hospitals kept up to date, the working man would recognise what was being done, and would contribute. He hoped State intervention in connection with hospitals would never come about: he had seen such intervention in connection with education, and the education had gone down instead of going up. State control would not be found so largely associated with personal sympathy, which was so very important in connection with the sick. He concluded by criticising the lack of proper provision for nurses under the Act.

Mr. Deacon, Royal Infirmary, Liverpool:—

Liverpool has made grants to hospitals for 50 years, and the city had power to appoint a representative on the hospital committee. The annual subscribers to the Infirmary liked them to get as much money as possible from wherever they could get it. It would be premature for this meeting to pass a resolution as the Act had been operative only six months, and it was the experience of many that it had not had any definitely deleterious effect on our hospitals; the voluntary contributions to his Infirmary had not been lessened. Societies would have to wait three years before they would know how their funds stood. Another reason for waiting was the widely different conditions which existed in various parts of the country. In his city the various friendly societies were meeting to discuss this question, and he believed it was likely that they would agree to make payments to hospitals. Anything required by the patient when he came out, such as a convalescent residence or appliances, should be deducted before a payment was made to the hospital.

Mr. J. P. Somers, Bushey Heath Cottage Hospital:—

I represent a cottage hospital. It is usual in small hospitals to make a charge to patients in accordance with what they are able to pay, ranging from nothing to 3*s.* 6*d.* to 10s. per week. Since the introduction of the Insurance Act the number of patients at his establishment had diminished, for cases no longer came in primarily for good nursing, but only when seriously ill or when an operation was required. Clause 12, Subsection (*c*) of the Act said, "Where there are no dependants, approved societies can pay if an agreement has been made between the hospital and the society or committee." He thought the words "or committee" included the County Committee, but the secretary of the County Committee said the hospitals should not have patients who had no dependants. As a fact, half their approved cases had no dependants; they were domestic servants and the like, and they had no power to enter into an agreement. He wrote to the various societies, and most of them refused to enter into an agreement; they said they would consider each case on its own merits. But when an urgent case, such as appendicitis, came in it had to be dealt with at once. He had written to the Commissioners concerning the circular quoted by Dr. Mackintosh, but had not yet received a reply, asking whether, when a patient came for immediate operation and there was no previous agreement, the approved society had power to pay the hospital. If so, why enter into an agreement?

Mr. J. S. Neil, Wolverhampton General Hospital:—

I hold the opinion that the question is not one of having more beds; there were sufficient beds in the general hospitals to treat most of the patients; the great want was money for maintenance. Since the introduction of the Insurance Act the patients in his hospital had increased twenty per cent. Still, he did not think this increase would continue, and neither the private donations nor the workmen's contributions had decreased. Still, it was now a great difficulty to get new people to contribute. He believed a time would come in this country—though it might not be for ten years—when patients under the Insurance Act would have to be provided for either by the Commissioners, the friendly societies, or by the municipalities. That did not necessarily mean State control. With reference to Sir Henry Burdett's suggestion for building new wings and making the receipts of the hospital the first charge, as the receipts of hospitals were already insufficient to meet the expenditure, he did not see how they could be regarded as security of any kind. He agreed as to the importance of providing hospital accommodation for other than the working classes—schoolmasters, schoolmistresses, clerks, and other educated people, who, however, had not much income, and yet did not like coming into a free ward and making use of charity. But paying establishments should be part of general hospitals, both on the grounds of convenience and economy of working.

Councillor Macpherson, Governor, Royal Infirmary, Edinburgh:—

I am fully in agreement with the idea that there should be pay departments in connection with the general voluntary hospitals. Many people used the voluntary hospitals without contributing to them at all, and very few gave in anything like the proportion of their indebtedness. He also agreed that it was as yet too soon to come to any resolution concerning the Insurance Act; but he thought the Council of the Association should meet at once and send a deputation to the House of Commons dealing with the amendments of the Act. It was of no use waiting until all the Insurance Committees throughout the country were able to submit the points they desired to see amended. The hospitals were faced with the danger of their work being paralysed by the cutting off of sources of income. Contributions to the Edinburgh Royal Infirmary had recently decreased, and the governors resolved to give notice that insured patients would not now be treated in the Royal Infirmary. That created a perfect furore; there was a lot of correspondence in the press. The various working-men's associations had banded themselves together and made contributions, in many cases the employers joining with them; but when the decision became known there were threatenings that these contributions would cease. The governors met, and decided to suspend any action for six months, until the working of the Act had become better known. At the last meeting the clerk stated that of the whole of the in-patients 42 per cent. were insured persons, while of the out-patients the percentage was much larger. Clearly, the hospitals must either cut off the insured patients, or else treat them at the public expense. He understood that under the Insurance Act there would be an allowance made for hospital as well as sanatorium treatment, but at present that did not seem to have taken shape. Some members would like that point brought before the Chancellor of the Exchequer. Some arrangement must be made, or the voluntary hospitals would go down. If the voluntary hospitals were to treat Insurance patients, they must be paid for their maintenance. The *raison d'être* of all hospitals was that the needy and suffering should have immediate attention.

Sir Henry Burdett explained that his suggestion was that the interest on the loan and sinking fund should be a charge on the *pro rata* payments received for the patients admitted into the new wards. But his view accorded with Mr. Neil's that it was desirable to provide for the treatment of patients in the pay-pavilions also. The hospital to which he referred had been going since 1880; it was rebuilt six years ago, and two-thirds of the money for that had already been repaid out of patients' payments.

Mr. Alvey, Charing Cross Hospital, London:—

I cannot speak for London hospitals generally, only for a part. Dealing with Sir Henry Burdett's suggestion as to a loan, he thought no hospital committee at the present time, which could lay claim to sanity, would attempt to borrow money for such a purpose as that to which Sir Henry referred; the object for which this money was to be borrowed was so problematical. He thought it would be some considerable time before the pressure on beds and the demand for more would come to the extent forecasted by Sir Henry Burdett. That was not the experience in London. The effect of the Insurance Act had been to decrease the number of out-patients, to diminish the constant out-patient attendances and casualties, and to keep away many trivial cases which hospitals were willing to do without. The serious cases still remained. The Act had made no difference to in-patients. And if such pressure as had been predicted should come about, as it would have been due to the Insurance Act it must be dealt with not by the voluntary hospitals, but by the Government. The chief problem at present was as to how to maintain the beds now existing. Mr. Carnt had shown that in most cases approved societies were willing to meet the hospitals fairly and squarely, and pay the sickness benefit wherever possible. He disagreed with any speaker who believed that any of the measures advocated would result in State control; he did not think approved societies would put forward any such claim. He was largely with Sir William Osler in thinking that the voluntary system would have to be considerably modified in the future not only on account of the Insurance Act, but because of the whole trend of things; all classes would have to be catered for by introducing some system of payments into the wards, while still retaining a free section. He believed the roots of the voluntary system were too deeply planted in the life of our community to be so rapidly devitalised as to justify it being pulled up root and branch.

Mr. Shaw, Southport Infirmary:—

I thank the President for his manly and straightforward speech. The question of pay-patients at his institution not only affected the hospital, but also was of moment to the honorary medical staff, and he described the methods adopted. The amendments now being mentioned were surely the outcome of the experience in the working of the Act, and it would be wise to place the collective experience of the Association before the Chancellor of the Exchequer.

Mr. H. J. Toulmin, St. Albans:—

It would help if members were to memorialise their various members of Parliament; it would be a pity if the chances now open to amend the Act were not taken. He was sure subscriptions would fall off under the present conditions.

In reply to questions, the President announced that there would be no resolution on the subject submitted to the meeting, but that the Council would watch events and at the proper time submit considered proposals as occasion may decide.

### THURSDAY: AFTERNOON SESSION. DISCUSSION ON SIR THOMAS OLIVER'S PAPER.

SIR WILLIAM OSLER, BART., M.D., PRESIDENT, IN THE CHAIR.

[Sir Thomas Oliver's paper appears on page 413.]

Dr. Ebenezer Duncan, Victoria Infirmary, Glasgow:—

As I have had experience as physician to a large general hospital, and in a similar capacity in two of Scotland's largest sanatoria, I have been able to form definite conclusions on the subject under debate. He agreed that it was impossible to keep tuberculosis out of our general hospitals: it was a protean disease, affecting every organ and tissue of the body. Many patients had the disease in an unrecognised form when entering the hospitals; some cases diagnosed as pneumonia or pleurisy were found to have active tubercle of the lung. And in the surgical wards, too, there must inevitably be many cases of tubercular invasion. He advocated that every hospital should have special accommodation for patients who were found to be tuberculous. They could then be made to do just as well as when sent to special sanatoria: he had had as good results under those conditions as he had in the sanatoria with which he was associated. He felt convinced that active tubercle, when the air was not fresh and rapidly changed, spread quickly and was definitely infectious; but when the ventilation was generous, when patients were not placed very near each other, and when proper precautions were adopted with regard to spitting, there was no infectivity. Generally, hospital patients were not seen for a sufficient time to enable the physician to be sure they had not the seeds of some such disease about them. The period of incubation of tubercle in some cases was a very prolonged one. The wording of the Insurance Act did not restrict tubercle to cases affecting the lung; any cases of the disease could be claimed for. He did not fear any harm from what might be done by the local authorities or by the Government. Government would not be eager to interfere so long as hospitals continued to be managed as well as at present; but he believed contributions to hospitals would suffer when the present wave of prosperity in the country had passed.

Mr. J. C. Buchanan, Metropolitan Hospital, London:—

My institution has taken more active steps than have some other hospitals in regard to the treatment of tuberculosis. He referred to the preliminary treatment of tuberculosis in London at dispensaries, which had been organised by a strong and well-informed body. There would be need for more of these dispensaries to be established in order to properly carry on the work; but the greatest need was for co-operation with the large general hospitals, which was encouraged by the action and by the courtesy of the London County Council. It was important that the patients who came to the dispensaries should have the advantage of the highest skilled attention, and no doubt that meant it should be at the hands of the medical staffs of the hospitals. The tuberculosis officers appointed must be trained by their own staffs, for it took a long time to produce a properly trained tuberculosis officer. There was also the question of organisation, and joining the hospitals up with the dispensaries would effect a great saving. The prescription of areas for certain hospitals was a difficulty, especially for such large hospitals as St. Bartholomew's; and, moreover, they could not cater for the important sphere of home-visiting. The latter, he thought, should be done by the medical officers of health, in connection with which authority there already existed a visiting service. It was most important, having prescribed for the patients, to follow them to their homes and see if the treatment was being properly carried out. And not until there were sufficient officers trained in the diagnosis of tubercle, should an attempt be made to treat such cases at institutions than at general hospitals. In his hospital, the public department had been closed, and offered to the Medical Officer of Health for the Borough of Hackney, and but for the intervention of the London County Council it would have been staffed by the hospital staff, and cases would have been visited in their homes. He proceeded to speak of the great benefit from sending patients to the Home at Cranbrook, and said there seemed, from what had been said, to be no need for the scare of residents and the depreciation of property, when the erection of a sanatorium was talked of. The Metropolitan Hospital possessed an island site, on which more accommodation could be erected, but funds were greatly needed; and he hoped to see increasing efforts made to rid the country of tuberculosis, in the same way as cholera and smallpox.

Mr. Fitzgerald, General Infirmary, Chester:—

My board applied to the Chester Insurance Committee for remuneration for treating tuberculous children under the sanatorium scheme, but were told none would be forthcoming for the treatment of surgical tuberculosis.

Dr. Brooks (Oxford):—

In my own opinion it is difficult to lay down formulæ as to future development, but one could run the idea of the infectiousness of tubercle to death; and if too strong a line in that regard were taken it would prevent much good work being done, and being done economically. Tuberculosis was such a widespread disease that it could not be removed entirely from the general hospital; moreover, the best treatment the patient could get was hospital treatment. He went to a discussion in connection with a general convalescent home, as to whether tuberculous patients should be received there or not. They had been taken for many years, and he believed they had been benefited. Then the infection scare arose, and the committee said they would have no more tuberculous cases there, as they might infect the other patients. He noticed on his way home—it was a winter's day—over 200 yards of Magdalen Bridge, some fifty patches of expectoration, a large proportion of which were probably tubercular. A large number of apparently healthy people walking about were tubercular. If one limited oneself to the infective stage, one missed the most important series of influences from the point of view of elimination—namely, the social. By ensuring to these early cases good food, more rest, and some easing of their work much good work would be done, and more economically than at a later stage. The Act had been in operation too short a time to permit of a good scheme having yet been evolved. The scheme at Oxford was to give facilities at the hospital for the working of the Insurance Act in regard to tuberculosis.

Mr. Blake, Royal Infirmary, Oldham:—

The staff of my hospital are in favour of the Act. Section 8, Subsection (*b*), provided for treatment in sanatoria of persons suffering from tuberculosis or such other diseases as the Local Government Board Act hereinafter called sanatorium benefit; and Section 16, Subsection 3, said the insured person should not be entitled to sanatorium benefit unless an Insurance Committee recommended the case for such benefit. The highest authority to whom they could appeal in Lancashire stated that the medical benefits and the sanatoria benefits were separate from each other. Some of the staff who were on a panel had had cases of tuberculous pleurisy; if they supplied such persons with medicine they could not be recouped for it. If that were true, surely the Act needed amendment.

Mr. Deacon, Liverpool:—

In the case of Liverpool the Corporation has undertaken the treatment of cases of tuberculosis whether insured or not. In his Infirmary there was an arrangement with the Corporation that cases fit for operation should be sent to the Infirmary, for which 27s. 6d. per week was paid. It was a temporary arrangement, as the whole matter would come up for consideration in July.

Mr. Fitzgerald (Chester) said the Chester Town Clerk informed them that the Commissioners limited the treatment to pulmonary tuberculosis.

Mr. Kemp, Charity Organisation Society, London:—

I know that in London insured people with tuberculosis of joints and bones are receiving treatment from the Insurance Committee. It had been his function to go round to many hospitals and dispensaries to see how they were doing their work, and how they proposed to do it under the new scheme which was promised in London under the London County Council. He spoke in detail of the Interim Report of the Departmental Committee on Tuberculosis, known as the Astor Report, the unanimous conclusions of which should carry great weight. The hospitals would naturally be used for consultations in difficult cases, and for the treatment of difficult cases when special apparatus was required. The Committee recommended that a certain amount of education should be given in the dispensaries, the most important feature of which was to get cases so early as to be really preventive centres, and the subsequent following up of the cases to their homes exercised a great check upon spread. Many felt that they had now got beyond the time when they could be satisfied with the ordinary out-patient departments for tuberculosis, for they were not in close enough touch with the patients. He did not think the London County Council suggested that every sufferer should be compelled to go to the dispensary. In Paddington and Marylebone, however, the dispensaries notified 44 per cent. and 59 per cent. respectively of the total notifications of the year, which showed they were doing their work efficiently. He was convinced that little real good would be accomplished in stamping out the disease until the very early cases were spotted and dealt with.

Mr. J. H. Cooke (Winsford) said that when he was at the Rockies. 300 Red Indians came to the hotel, and he was told they had 50,000 square miles to themselves and yet they were dying of consumption. So it was not simply a question of having plenty of air; their sanitary arrangements were very bad.

Sir Henry Burdett:—

It is unfortunately true that the tuberculin dispensaries and some County Council clinics as at present organised are not efficient. Wherever these institutions were established there should be an arrangement for their continuous inspection; and care should be taken that the hours fixed for patients to attend should see all the staff punctually present. It is a grave wrong to the poor to fix 2 o'clock in the afternoon for the patients to be there, but no medical man in attendance until 3.30. Again, the practitioners appointed to administer anæsthetics should have had proper experience of the work, the failure to ensure which had sometimes led to results which he could not speak of there. The practitioners appointed to do the work should be those who had that special knowledge, which would result in their work being efficient.

The Rev. G. B. Cronshaw, Radcliffe Infirmary, Oxford:—

I may give an account of the work which has been attempted at Oxford. The Radcliffe Infirmary was the only hospital within twenty miles, and its work was somewhat limited by the means of conveyance to the hospital. An effort had been made to avoid the errors which had been detailed to the meeting. The grand basis of the work was the prevention of tuberculosis—while relieving the distress they were trying hard to remove the cause; they were taking a wide survey of the disease. The hospital allowed the city to use its out-patient department at the times which best suited it; and the tuberculosis officer kept his case-notes there and saw his patients there. He was likewise afforded facilities to use the special departments of the hospital for suitable cases. He felt that if hospitals did not permit this sort of thing they were betraying a sacred trust, for after all they were trustees for the public health. The people would still have had the use of all the hospital facilities even if there had been no Insurance Act. The agreement between the city and the hospital was for one year only, and renewable from year to year. There was such close co-operation that there was an absence of friction between the preventive side and the hospital management. The City Council had, as an experiment, taken six beds at 30s. a week for tuberculous patients, and the Council would probably give some £150 in addition.

Mr. Lupton, Leeds:—

I would like to direct attention to the training of the medical student, which came into the work of the larger hospitals which had a medical school attached. He emphasised the fact that it was the duty of all such hospitals to take a share in this tuberculosis work; otherwise medical officers would arise who did not properly understand the disease.

Sir William Osler:—

The President, in closing the discussion, expressed the indebtedness of the Association to Sir Thomas Oliver for his instructive paper. He (Sir William) had for many years taken a close interest in tuberculosis and its association with general hospitals. From the standpoint of the wards, practically all forms of tuberculosis could be safely admitted except the advanced open cases. Miliary tuberculosis, acute pleurisy, acute tuberculous peritonitis, tuberculous meningitis had to be admitted to general hospitals; there was nowhere else for them to go to; and there were suspected cases which came in for observation. Even early cases of tuberculosis had been successfully treated on the balconies of Radcliffe Infirmary. He claimed that the dispensary of a general hospital for tuberculosis might be made ideal. It was much more satisfying to have the organisation and the equipment in connection with a general hospital. In the first place one had the atmosphere for the men working there, and in the second it was much better and easier for the patients, for they could be transferred from one department to another as needed. There was no social work so thoroughly well done as in certain of the best organised tuberculosis dispensaries connected with general hospitals; he could name hospitals where about a dozen nurses were deputed to that particular social work. That work done in connection with St. Thomas's Hospital, for instance, had brought out the fact that if tuberculosis was to be headed off it must be largely a matter of having proper housing and right methods of living. The work in the general hospitals, the teaching hospitals, and the dispensaries should be closely linked up, and be of the best type, so as to do the best possible in prevention, cure, and education. In the dispensary he started at, the Johns Hopkins Hospital, every medical student of the school passed through the dispensary as part of his routine clinical work.

